# Salivary Microbiota and Host-Inflammatory Responses in Periodontitis Affected Individuals With and Without Rheumatoid Arthritis

**DOI:** 10.3389/fcimb.2022.841139

**Published:** 2022-03-14

**Authors:** Kaja Eriksson, Anna Lundmark, Luis F. Delgado, Yue O. O. Hu, Guozhong Fei, Linkiat Lee, Carina Fei, Anca I. Catrina, Leif Jansson, Anders F. Andersson, Tülay Yucel-Lindberg

**Affiliations:** ^1^ Department of Dental Medicine, Division of Pediatric Dentistry, Karolinska Institutet, Huddinge, Sweden; ^2^ KTH Royal Institute of Technology, Science for Life Laboratory, Department of Gene Technology, School of Engineering Sciences in Chemistry, Biotechnology and Health, Stockholm, Sweden; ^3^ Department of Microbiology, Tumor and Cell Biology, Centre for Translational Microbiome Research, Karolinska Institutet, Stockholm, Sweden; ^4^ Center for Rheumatology, Academic Specialist Center, Stockholm Health Region, Stockholm, Sweden; ^5^ Rheumatology Clinic, Karolinska University Hospital, Stockholm, Sweden; ^6^ Department of Dental Medicine, Division of Periodontology, Karolinska Institutet, Huddinge, Sweden; ^7^ Department of Periodontology, Folktandvården Stockholms län AB, Folktandvården Eastmaninstitutet, Stockholm, Sweden

**Keywords:** periodontitis, rheumatoid arthritis, microbiota, saliva, host inflammatory mediators, cytokines, machine learning

## Abstract

**Objectives:**

Periodontitis and rheumatoid arthritis (RA) are two widespread chronic inflammatory diseases with a previously suggested association. The objective of the current study was to compare the oral microbial composition and host´s inflammatory mediator profile of saliva samples obtained from subjects with periodontitis, with and without RA, as well as to predict biomarkers, of bacterial pathogens and/or inflammatory mediators, for classification of samples associated with periodontitis and RA.

**Methods:**

Salivary samples were obtained from 53 patients with periodontitis and RA and 48 non-RA with chronic periodontitis. The microbial composition was identified using 16S rRNA gene sequencing and compared across periodontitis patients with and without RA. Levels of inflammatory mediators were determined using a multiplex bead assay, compared between the groups and correlated to the microbial profile. The achieved data was analysed using PCoA, DESeq2 and two machine learning algorithms, OPLS-DA and sPLS-DA.

**Results:**

Differential abundance DESeq2 analyses showed that the four most highly enriched (log2 FC >20) amplicon sequence variants (ASVs) in the non-RA periodontitis group included *Alloprevotella* sp., *Prevotella* sp., *Haemophilus* sp., and *Actinomyces* sp. whereas *Granulicatella* sp., *Veillonella* sp., *Megasphaera* sp., and *Fusobacterium nucleatum* were the most highly enriched ASVs (log2 FC >20) in the RA group. OPLS-DA with log2 FC analyses demonstrated that the top ASVs with the highest importance included *Vampirovibrio* sp. having a positive correlation with non-RA group, and seven ASVs belonging to *Sphingomonas insulae*, *Sphingobium* sp., *Novosphingobium aromaticivorans*, *Delftia acidovorans*, *Aquabacterium* spp. and *Sphingomonas echinoides* with a positive correlation with RA group. Among the detected inflammatory mediators in saliva samples, TWEAK/TNFSF12, IL-35, IFN-α2, pentraxin-3, gp130/sIL6Rb, sIL-6Ra, IL-19 and sTNF-R1 were found to be significantly increased in patients with periodontitis and RA compared to non-RA group with periodontitis. Moreover, correlations between ASVs and inflammatory mediators using sPLS-DA analysis revealed that TWEAK/TNFSF12, pentraxin-3 and IL-19 were positively correlated with the ASVs *Sphingobium* sp., *Acidovorax delafieldii*, *Novosphingobium* sp., and *Aquabacterium* sp.

**Conclusion:**

Our results suggest that the combination of microbes and host inflammatory mediators could be more efficient to be used as a predictable biomarker associated with periodontitis and RA, as compared to microbes and inflammatory mediators alone.

## Introduction

Periodontitis is a microbial-induced chronic inflammatory condition, affecting tooth-supporting structures eventually resulting in tooth loss (Page and Kornman, 1997; Darveau, 2010). The disease is highly prevalent, and it has been estimated that 42% of the adult population are affected (Eke et al., 2020). Severe form of periodontitis, afflicting 8-11% of the population (Kassebaum et al., 2014; Eke et al., 2020) not only cause tooth loss, but can also increase the risk for chronic disorders, such as cardiovascular disease, diabetes, and RA (Hajishengallis, 2015; Hajishengallis and Chavakis, 2021), and may have a role in several other systemic diseases and conditions such as obesity, pancreatic cancer, and Alzheimer’s disease (Falcao and Bullon, 2019). The process of periodontitis is initiated when the biofilm forms in proximity to the gingiva and releases various substances, such as lipopolysaccharide, peptidoglycans and toxins, triggering host response (Page and Kornman, 1997; Pollanen et al., 2012; Yucel-Lindberg and Bage, 2013). As a consequence of bacterial challenge, the host immune response initiates a cascade of reactions including stimulation of pro-inflammatory cytokines, chemokines, prostaglandins, toll-like receptors and numerous proteolytic enzymes, collectively contributing to the pathogenesis of periodontitis (Bascones et al., 2005). Thus, the ongoing “battle” of inflammation is not only measurable locally in the oral samples, but also systemically as increased levels of pro-inflammatory mediators have been demonstrated in the blood and saliva samples of patients with periodontitis (Van Dyke, 2009; Tasoulas et al., 2016).

The painful and debilitating systemic inflammatory disease RA, affects almost 1% of the world population, predominantly women (Scott et al., 2010; Gerlag et al., 2016). It is associated with long term morbidity and early mortality despite anti-rheumatic treatment (Gerlag et al., 2016). This chronic autoimmune disorder has also been associated with systemic complications as well as cardiovascular comorbidity and type 1 diabetes (McInnes and Schett, 2011; Radner et al., 2017; Smolen et al., 2018). In addition, an association between RA and periodontitis has been suggested and significantly higher risk of periodontitis has been observed in patients with RA compared to healthy (non-RA) controls using meta-analysis investigating the relationship between periodontitis and RA (Fuggle et al., 2016).

Periodontitis and RA share numerous etiological and pathogenic features including tissue and bone destruction, production of inflammatory mediators including cytokines, prostaglandins and tissue degrading enzymes (e.g., matrix metalloproteinases) and common risk factors such as smoking (Rosenstein et al., 2004; de Pablo et al., 2009). Epidemiological studies suggest an association between periodontitis severity and RA disease activity based on increased incidence of periodontitis in patients with RA and a dose-response pattern (de Pablo et al., 2009; Punceviciene et al., 2021). The confirmed link between RA and periodontitis is hypothesized to be mediated by oral microbiota involving the periodontitis-associated pathogens *Porphyromonas gingivalis* and *Aggregatibacter actinomycetemcomitans* due to citrullination process (Smolen and Steiner, 2003; Rosenstein et al., 2004; Kharlamova et al., 2016; Konig et al., 2016). In addition, the oral bacteria, *Cryptobacterium curtum*, another pathogen capable of producing citrulline (Uematsu et al., 2006), was found to be more abundant in periodontally healthy patients with RA and enriched in the oral microbiome of early RA patients (Scher et al., 2014; Lopez-Oliva et al., 2018).

Previously, we have analysed the oral microbial profiles of subjects with RA in subgingival plaque and saliva samples using 16S rRNA gene sequencing. When investigating the microbial profiles of patients with RA in relation to periodontal status, we found that patients with moderate/severe periodontitis had significantly higher abundance of *Desulfobulbus* sp., *Prevotella* sp., *Bulleidia* sp., *Capnocytophaga* sp., and *Tannerella forsythia* in plaque, when compared to RA with no/mild periodontitis (Eriksson et al., 2019). In addition, in saliva samples of non-RA subjects with periodontitis, we recently reported the microbes *Eubacterium saphenum*, *Tannerella forsythia*, *Filifactor alocis*, *Streptococcus mitis/parasanguinis*, *Parvimonas micra*, *Prevotella* sp., *Phocaeicola* sp., and *Fretibacterium* sp. to be more abundant in periodontitis, compared to healthy controls (Lundmark et al., 2019). In the current discovery-driven study, we aimed to profile and compare the oral microbial composition and its host-inflammatory mediator profile in periodontitis affected individuals, with and without RA, as well as to predict biomarkers, of bacterial pathogens and/or inflammatory mediators associated with periodontitis and RA.

## Materials and Methods

### Ethics Approval

This study was approved by the Regional Ethical Review Board in Stockholm (2009/792-31/4, 2014/1588 – 32/3 and 2015/766-32) and included an informed written consent from all participants.

### Study Design, Participants, and Periodontal Examination

A total of 101 participants, all from the clinics in the Stockholm County area, were included in the current study population, 53 subjects with RA and chronic periodontitis and 48 non-RA with chronic periodontitis. The participants were recruited continuously between the years 2015-2020, and the patients with RA consisted of 46 females and 7 males, aged 60.4±12.2 years. The non-RA subjects with periodontitis consisting of 26 females and 22 males, had a mean age of 61.2±13.6 years.

Patients with RA (mean disease duration 10.3 years; min-max 1-37 years), all fulfilling the 2010 American College of Rheumatology (ACR) Criteria for RA (Aletaha et al., 2010) were recruited from Karolinska University Hospital in Solna and Huddinge (Stockholm, Sweden). All subjects with chronic arthritis underwent a full mouth dental and periodontal examination performed by a single dentist (KE) calibrated by a periodontist (LJ) as previously described (Eriksson et al., 2019). The patients did not undertake periodontal treatment for 3 months prior to the examination. Exclusion criteria also included other forms of arthritis, the use of antibiotics 3 months prior to the dental examination, pregnancy, and lactation.

Periodontitis was diagnosed based on measurements of clinical attachment loss (CAL) ≥5 mm, probing pocket depth (PPD) ≥4 mm, bleeding on probing (BoP) >30% as well as radiographic examinations. In both groups, the PPD ranged between 4-12 mm. With regard to periodontitis severity, 8% of RA and 15% of non-RA participants had at least one site with PPD ≥ 10 mm, indicating a severe form of chronic periodontitis, a statistically non-significant difference between the groups. The majority of the participants (92% RA and 85% non-RA) had PPD ≥ 4 mm and/or CAL ≥ 5/loss of supporting tissues exceeding 1/3 of the root length. Among RA participants, eight patients had cardiovascular disease, eight temporomandibular joint, seven high blood pressure, two diabetes, six gastrointestinal disorder and five had asthma. Among non-RA participants, three individuals had diabetes, nine individuals had high blood pressure and two cardiovascular diseases.

### Collection and Processing of Saliva Samples

In addition to the periodontal examination, samples of saliva were also collected for multiplex and 16S rRNA sequencing analysis. For the purpose of this study, stimulated saliva was collected since chewing may release bacteria from the gingival sulcus making the stimulated saliva collection method more appropriate for detecting periodontal pathogens, which has also been previously suggested (Belstrom et al., 2017). The participants were not allowed to eat or drink for an hour prior to saliva collection, according to previous protocols (Lim et al., 2017). Stimulated saliva was accumulated by each participant chewing on paraffin wax (1gram, Ivoclar Vivadent, Liechtenstein) for a duration of 2 min, and the samples were stored in sterile 50 mL falcon tubed and immediately frozen at −20°C. The collected samples were kept frozen until processing, followed by centrifugation at 500 x g for 10 min at 5°C and supernatants collected and stored in Eppendorf tubes at -80°C until further processing and analysis (Lundmark et al., 2017). Prior to DNA extraction and analysis, 400 µL of each saliva sample was centrifuged at 10,000 rpm for 15 min, and the pellets resuspended in 200 µL of PBS.

Total protein concentration in the samples was assessed by using the *DC*™ (detergent compatible) protein assay (Bio-Rad, Hercules, CA, USA) according to the manufacturer´s recommendations. Bovine serum albumin (Sigma-Aldrich, St. Louis, Missouri, USA) was used for preparing a standard curve and absorbance reading was measured at 690 nm with a microplate reader (Multiskan MS Type 352, Labsystems, Finland).

### DNA Extraction

Bacterial DNA was isolated from the centrifuged and resuspended saliva pellets by using a DNA extraction kit (QiaAmp DNA Mini Kit, QIAGEN, Sweden) (Lundmark et al., 2019), following the instructions of the manufacturer. In brief, the samples containing 200 µL of PBS (described above) were resuspended in tissue lysis buffer and lysed using proteinase K (QIAGEN) at 56°C (10 min), purified using ethanol-containing buffers, and eluted in 50 μL nuclease-free water, according to manufacturer’s protocol. The amount of DNA in each sample was quantified by Qubit™ 2.0 fluorometer (Invitrogen, Life Technologies, USA).

### Generation of 16S rRNA Gene Libraries and Sequencing

The variable regions (V3-V4) of the 16S rRNA gene were amplified from 2.0 ng DNA from each saliva sample. The final concentrations of the PCR reactions were 1x KAPA HotStart ReadyMix (KAPA Biosystems, Wilmington, MA, USA), 1.0 µM forward primer (5’- CCTAHGGGRBGCAGCAG-3’), 1.0 µM reverse primer (5’-GACTACHVGGGTATCTAATCC-3’) and 0.1 ng/µl DNA. The PCR conditions during the amplification were 98°C for 2 min followed by 26 cycles of 98°C for 20 s, 54°C for 20 s and 72°C for 15 s, and a final elongation step of 72°C for 2 min. After amplification the samples were purified with Polyethylene Glycol 6000 (Merck Millipore, Darmstadt, Germany) and carboxylic acid beads (Dynabeads^®^ MyOne™, Thermo Fisher Scientific, Waltham, MA, USA) according to the procedure described by Lundin and colleagues (Lundin et al., 2010). The libraries were indexed using 12 µL of each purified sample, 0.4 µM forward and 0.4 µM reverse indexing primer and KAPA HotStart ReadyMix. The PCR conditions were 98°C for 2 min followed by 10 cycles of 98°C for 20 s, 62°C for 30 s, and 72°C for 30 s, and a final elongation step of 72°C for 2 min. After amplification the samples were quantified using Qubit™, diluted to 2.0 ng/μl and the indexed samples were purified again, in a similar manner as described above. Equimolar amounts of indexed samples were mixed, and the two pools of samples created were sequenced in separate runs on the Illumina MiSeq platform (Illumina Inc, San Diego, CA, USA) at NGI/SciLifeLab Stockholm.

After sequencing, five samples were excluded due to low number of reads (cut-off value 10,000 reads), which resulted in inclusion of 52 samples from the cohort of patients with RA and 45 samples from individuals without RA. The median depth of sequencing, after exclusion of low-depth libraries, was 195,300 reads per sample (Interquartile range (IQR): 146,700 – 218,600 reads).

### Sequence Data Processing

The DADA2 pipeline (Caporaso et al., 2010) was used to process the amplicon reads. First, low-quality reads and primers were trimmed and filtered. Remaining reads were dereplicated, the sequence variants were inferred, and the paired-end reads were merged by requiring 30 bp overlap with no mismatches. Thereafter, two sequencing runs were merged, a sequence table was constructed, followed by removal of chimeras. The reads were taxonomically assigned using RDP training set 14 (Cole et al., 2014). A phylogenetic tree was constructed using the scripts align_seqs.py, filter_alignment.py, and make_phylogeny.py, as provided by QIIME (Caporaso et al., 2010). The sequence table, taxonomic assignment, and phylogenetic tree were used to create a phyloseq object (McMurdie and Holmes, 2013), which was used in all subsequent analyses.

### Analysis of Sequence Data

The analysis identified in total 12,371 taxa. Sequence variants only present in 5% or less of the samples were filtered out, which resulted in 1,162 taxa included in the final dataset. Analysis of differential abundance of microbes between saliva samples from periodontitis patients with and without RA was performed using DESeq2 (Love et al., 2014). A bar plot depicting the significantly differentially abundant taxa was constructed in ggplot2 (Wickham, 2016). All statistical analyses were performed using R, version 3.6.1 (R Core Team, 2015).

Principal Coordinates Analysis (using the function ordinate of the R package phyloseq (McMurdie and Holmes, 2013) was performed on unweighted UniFrac distances with taxa having a coefficient of variation >3.0. The Orthogonal Partial Least Squares Discriminant Analysis (OPLS-DA) classification, a machine learning algorithm, was performed on the relative abundances of amplicon sequencing variants, ASVs, (after mean-centering and unit variance scaling) using the ropls package (Thevenot et al., 2015) in R.

To evaluate the predictive performance of the OPLS-DA model, the model was first trained on a randomly selected subset of the samples (75% of samples), then validated on the validation subset (25% of samples). Next, Receiver Operating Characteristic (ROC), and Area Under the Curve (AUC) were calculated using the R package pROC (Robin et al., 2011). Additionally, the predictive power of the trained model was also evaluated using 7-fold cross-validation (default parameter of the OPLS function, R package ropls). The OPLS-DA model variable importance scores were extracted (using the function getVipVn in R package ropls) and visualized together with the log2 fold changes (log2 FC) of the ASVs.

### Determination of Levels of Inflammatory Mediators in Saliva Samples

The levels of inflammatory mediators in saliva samples were analysed using a commercially available, broad immunoassay multiplex kit panel (Human Inflammation panel) containing various detectable inflammatory mediators in the saliva, including members of the tumor necrosis factor α (TNF-α) superfamily (Bio-Rad Laboratories, Hercules, CA, USA). The measured analytes were: APRIL (also known as tumor necrosis factor ligand superfamily member 13 (TNFSF13), B-cell activating factor (BAFF), also known as TNFSF13B, soluble cluster of differentiation 163 (sCD163), glycoprotein 130 (gp130), also known as soluble interleukin 6 receptor b (sIL-6Rb), interferon alpha 2 (IFN-α2), sIL-6Ra, IL-8, IL-10, IL-19, IL-22, IL-35, matrix metalloproteinase 1 (MMP-1), pentraxin-3, sTNF-R1, sTNF-R2, thymic stromal lymphopoietin (TSLP), and TNF-like weak inducer of apoptosis (TWEAK) also known as TNFSF12.

### Statistical Analysis

All statistical analyses were conducted in R, version 3.3.3 (R Core Team, 2015). Significances of differences in levels of inflammatory mediators in saliva samples from periodontitis patients with RA compared to periodontitis patients without RA were assessed using the Wilcoxon rank sum test. False discovery rate (FDR) was used for p-value correction upon multiple comparisons using the Benjamini-Hochberg method (Benjamini and Hochberg, 1995). For dichotomous variables Fisher’s exact test was used.

### Correlation Analysis Between Microbiota and Inflammatory Mediator Levels

To investigate the correlations between microbiota and various host-mediated inflammatory mediators such as cytokines, the multivariate method sparse partial least squares discriminant analysis (sPLS-DA), implemented in the mixOmics R package (Rohart et al., 2017) was used to perform integration (function block.splsda) and visualization (function circosPlot) of microbial and cytokine data. The cytokine data and the normalized (using function counts in DEseq2 R package) (Love et al., 2014) relative abundances of the ASV data were scaled to zero mean and unit variance prior to analysis with sPLS-DA.

## Results

### Composition of the Salivary Bacterial Communities

**Figure 1:**
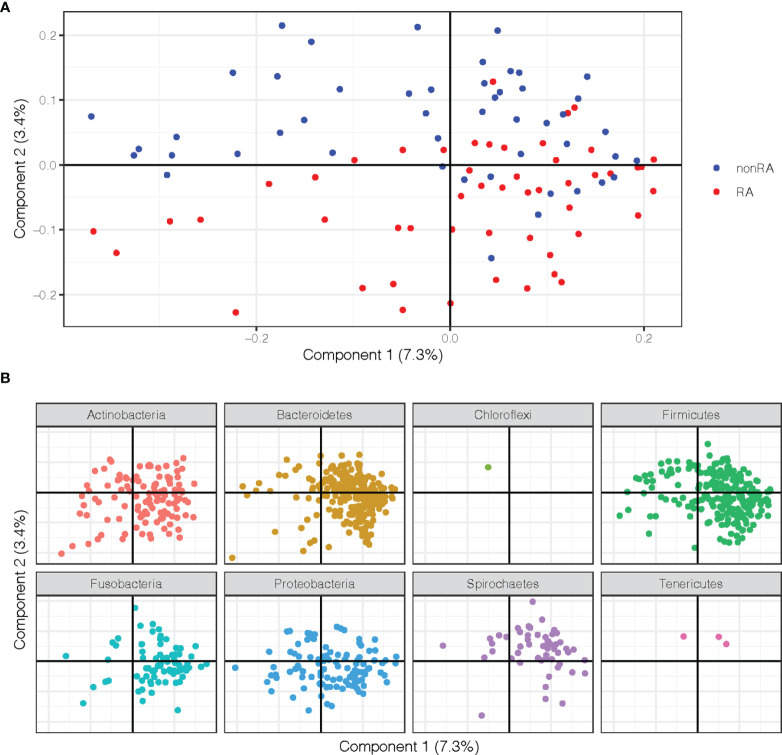
Principal Coordinate Analysis (PCoA) plots on the first two coordinates with UniFrac distances. Variation explained by the coordinates are given within parenthesis. **(A)** A sample projection plot with the samples color-coded by disease status. Saliva samples from patients with RA and periodontitis are highlighted in red and periodontitis patients without RA are in blue. **(B)** The contribution of ASVs are visualized in the same PCoA space as the samples and shaded according to their phylum assignment.

To characterize similarities or differences in the composition of bacterial communities between samples from periodontitis patients with and without RA, Principal Coordinate Analysis (PCoA) was applied on UniFrac distances generated for the microbial data. As shown in **Figure 1A**, the PCoA separated the samples of patients with RA and those without RA (controls) along component 2, indicating that the second largest variation in the dataset is dependent on the RA status.

In order to further explore the underlying rationale for this separation, the ASVs were instead visualized in the PCoA and shaded by phylum, demonstrating that several phyla also separated along the second component. These analyses revealed that phyla Spirochaetes, Tenericutes, and Chloroflexi were more abundant in the upper part of the plot and therefore were more predominant in samples from the non-RA group, which also clustered to the top (**Figures 1A, B**). The phyla Actinobacteria, Bacteriodetes, Proteobacteria, and Firmicutes, on the other hand, were more abundant in the bottom part of the plot, in the same area as the RA samples (**Figures 1A, B**).

### Differentially Abundant Taxa in Periodontitis Patients With and Without RA

**Figure 2:**
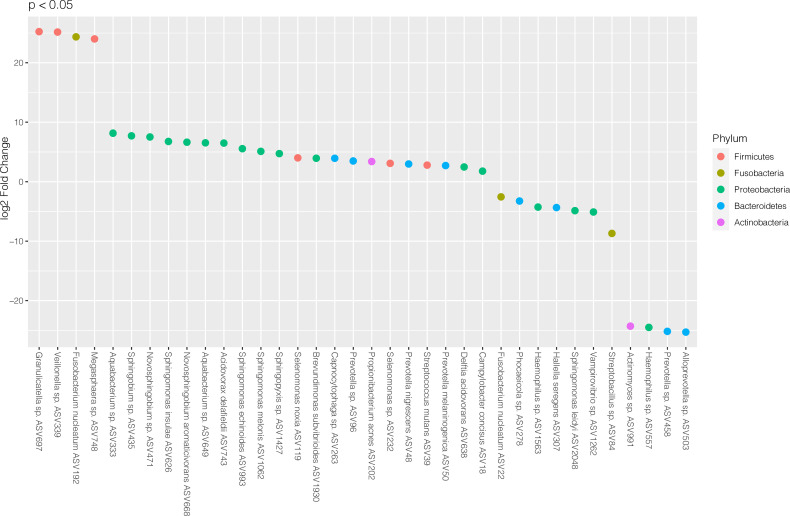
Differentially abundant amplicon sequence variants (ASVs) identified through DESeq2 testing. Each ASV is shown as its lowest annotated taxonomic rank together with its ASV ID. The ASVs are color-coded according to the phyla they belong to and plotted according to their log2 fold change (log2 FC), calculated as the levels in samples from patients with RA and periodontitis relative the levels in samples from periodontitis patients without RA, with size factor normalization as implemented in DESeq2.

To investigate which microbes differed in abundance between saliva samples from periodontitis patients with RA compared to periodontitis patients without RA, differential abundance analyses were performed using DESeq2. The analysis revealed significant (FDR adjusted p < 0.05) alterations in 36 ASVs as demonstrated in **Figure 2**. Among these, 25 ASVs were significantly (FDR adjusted p < 0.05) more abundant in saliva samples from patients with periodontitis and RA, whereas 11 ASVs were more abundant in periodontitis patients without RA.

In the RA group, the ASVs that were most highly enriched (log2 FC > 20) included three from Firmicutes phylum; *Granulicatella* sp. (ASV697), *Veillonella* sp. (ASV339) and *Megasphaera* sp. (ASV748), and one from Fusobacteria phylum; *Fusobacterium nucleatum* (ASV192). In the non-RA group, the top four ASVs that were more highly abundant included two from Bacteroidetes phylum; *Alloprevotella* sp. (ASV503) and *Prevotella* sp. (ASV458), one from Proteobacteria phylum; *Haemophilus* sp. (ASV557) and one from Actinobacteria; *Actinomyces* sp. (ASV991) (**Figure 2**).

### Quantification of Salivary Inflammatory Mediators

**Figure 3:**
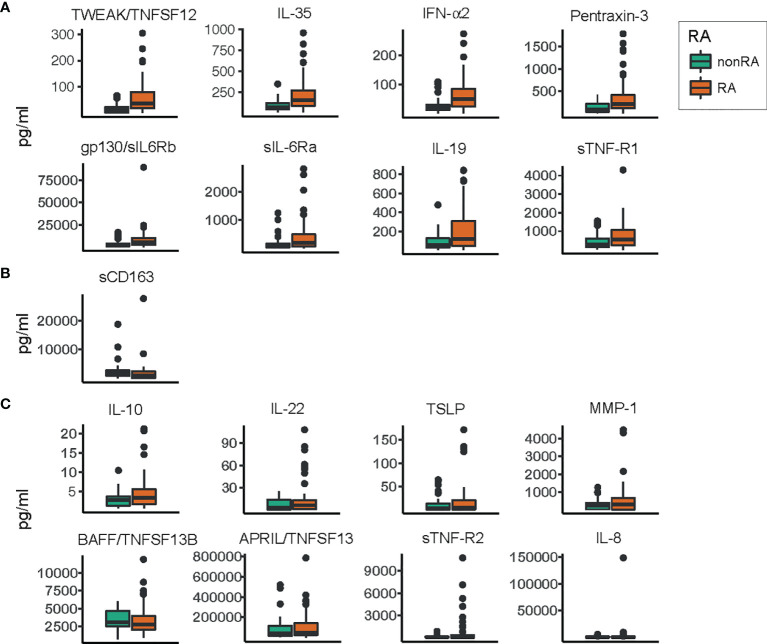
Levels of inflammatory mediators/cytokines in saliva samples from patients with and without RA. Box plots describing the levels of inflammatory mediators in saliva samples from periodontitis patients with RA and saliva samples from non-RA patients with periodontitis. Orange boxes represent the group of periodontitis patients with RA, and green boxes represent the group without RA. **(A)** Inflammatory mediators significantly (FDR adjusted p<0.05) enriched in saliva samples from patients with RA compared to non-RA patients. **(B)** The levels of soluble cluster of differentiation 163 (sCD163), the only inflammatory mediator significantly (FDR adjusted p<0.05) depleted in saliva samples from patients with RA compared to patients without RA. **(C)** Inflammatory mediators whose levels did not differ significantly between the two groups. All values are pg/ml, and within each group **(A–C)** the inflammatory mediators are ordered according to decreasing significance. TWEAK, tumor necrosis factor-like weak inducer of apoptosis; TNFSF12, tumor necrosis factor ligand superfamily member 12; IL, interleukin; IFN-α2, interferon alpha 2; gp130, glycoprotein 130; sIL-6Rb, soluble IL-6 receptor b; sTNF-R1, soluble TNF receptor 1; TSLP, thymic stromal lymphopoietin; MMP-1, matrix metalloproteinase 1; BAFF, B-cell activating factor; APRIL, a proliferation-inducing ligand.

The levels of 17 detectable inflammatory mediators were determined in the saliva samples using a multiplex bead assay and compared across periodontitis patients with and without RA (**Figure 3**). The analysis revealed that the levels of nine inflammatory mediators were altered between the two groups. Eight inflammatory mediators were present at significantly (FDR adjusted p < 0.05) higher levels in the samples from patients with RA, including TWEAK/TNFSF12, IL-35, IFN-α2, pentraxin-3, gp130/sIL6Rb, sIL-6Ra, IL-19, and sTNF-R1. The levels of these up-regulated inflammatory mediators in saliva samples from periodontitis patients with RA, compared to periodontitis patients without RA are demonstrated in **Figure 3A**.

One inflammatory mediator, sCD163, was present at significantly (FDR adjusted p < 0.05) lower levels in saliva samples from patients with RA and periodontitis, compared to patients with periodontitis without RA (**Figure 3B**). The rest of the investigated inflammatory mediators were not significantly altered. These included IL-10, IL-22, TSLP, MMP-1, BAFF/TNFSF13B, APRIL/TNFSF13, sTNF-R2, and IL-8, demonstrated in **Figure 3C**.

### Classification of Samples From Periodontitis Patients With and Without RA-Based on ASVs

Next, we used an OPLS-DA model to investigate whether the relative microbial ASVs abundances observed in the saliva samples could be used to discriminate individuals with RA from those without RA. We first trained the OPLS-DA model with data from 74 samples: 36 from patients with RA and 38 samples from non-RA patients. On this training dataset, we applied cross-validation to evaluate the predictive power of the model. Although the model fitted well to the training data set (R2Y value 0.931), the predictive performance of the model was low (Q2Y value 0.344, **Supplementary Figure 1A**). To further evaluate the predictive power of the model and to validate this model, we used the remaining 25 samples (not used during model training) consisting of 15 patients with RA and 10 patients without RA. The model classified these 25 samples into 12 RA and 8 non-RA subjects with high accuracy (0.8) and an AUC of 0.94.

**Figure 4:**
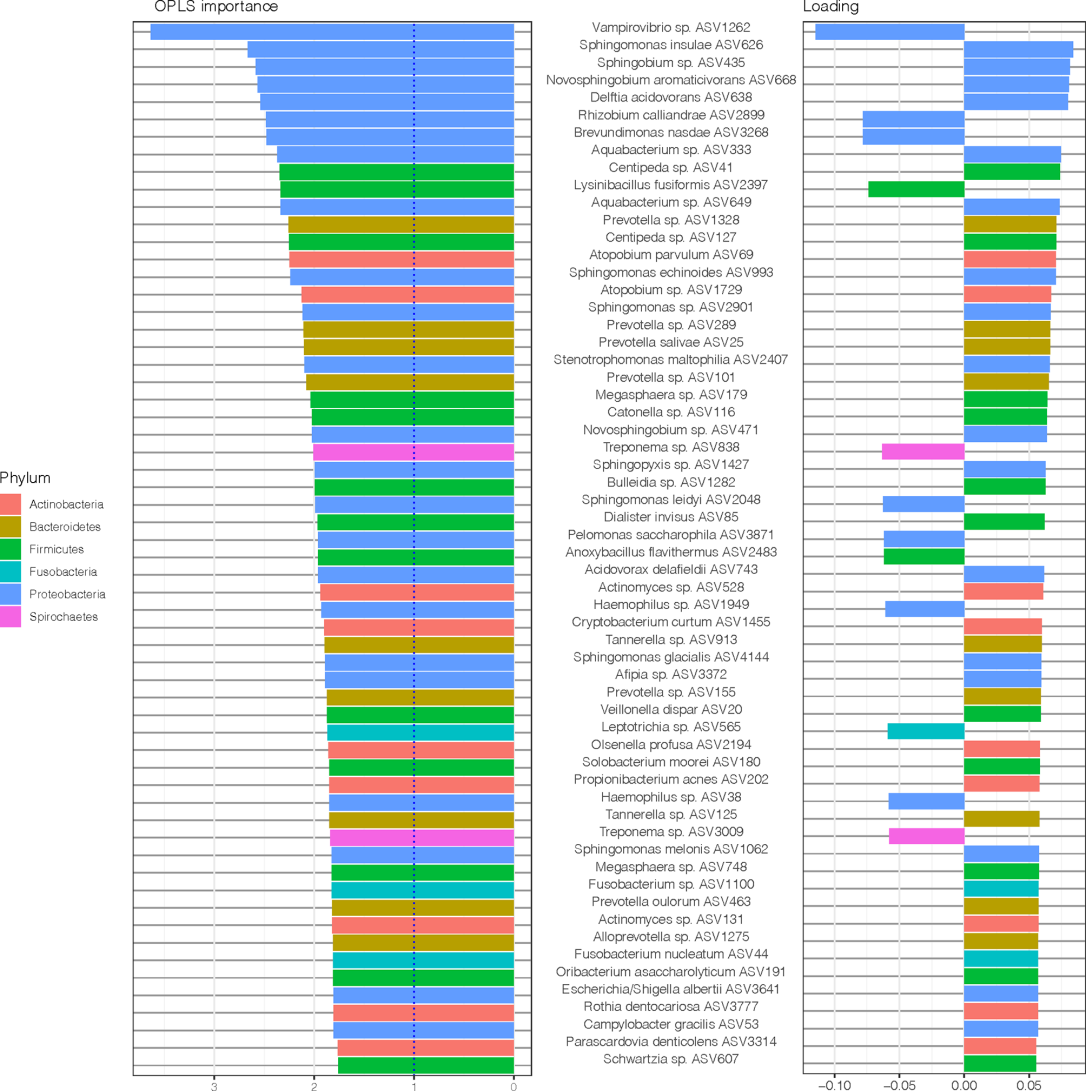
Amplicon sequences variants (ASVs) identified by Orthogonal Partial Least Squares Discriminant Analysis (OPLS-DA) analysis. In OPLS-DA, a single component serves as a predictor for the class. The sixty most important variables (with VIP > 1) for the classification of saliva samples from patients with and without RA are shown. The variable loading on the predictive component is shown (positive = discriminatory direction RA, negative = discriminatory direction non-RA). The lowest annotated taxonomic rank is shown for each ASV as well as the ASV IDs used for the analysis.

The most important ASVs for the classification of samples e.g., those with variable importance in projection (VIP) > 1 (Thevenot et al., 2015) and the variable loading on the predictive component is visualized in **Figure 4**. Among the top 15 ASVs with the highest importance (67% belonging to Proteobacteria phylum), four ASVs (*Vampirovibrio* sp. ASV1262, *Rhizobium calliandrae* ASV2899, *Brevundimonas nasdae* ASV3268 and *Lysinibacillus fusiformis* ASV2397) had a negative loading (positive correlation with non-RA group), whereas 11 ASVs (*Sphingomonas insulae* ASV626, *Sphingobium* sp. ASV435, *Novosphingobium aromaticivorans* ASV668, *Delftia acidovorans* ASV638, *Aquabacterium* sp. ASV333, *Centipeda* sp. ASV41, *Aquabacterium* sp. ASV649, *Prevotella* sp. ASV1328 *Centipeda* sp. ASV127, *Atopobium parvulum* ASV69, and *Sphingomonas echinoides* ASV993) had a positive loading (positive correlation with RA group).

**Figure 5:**
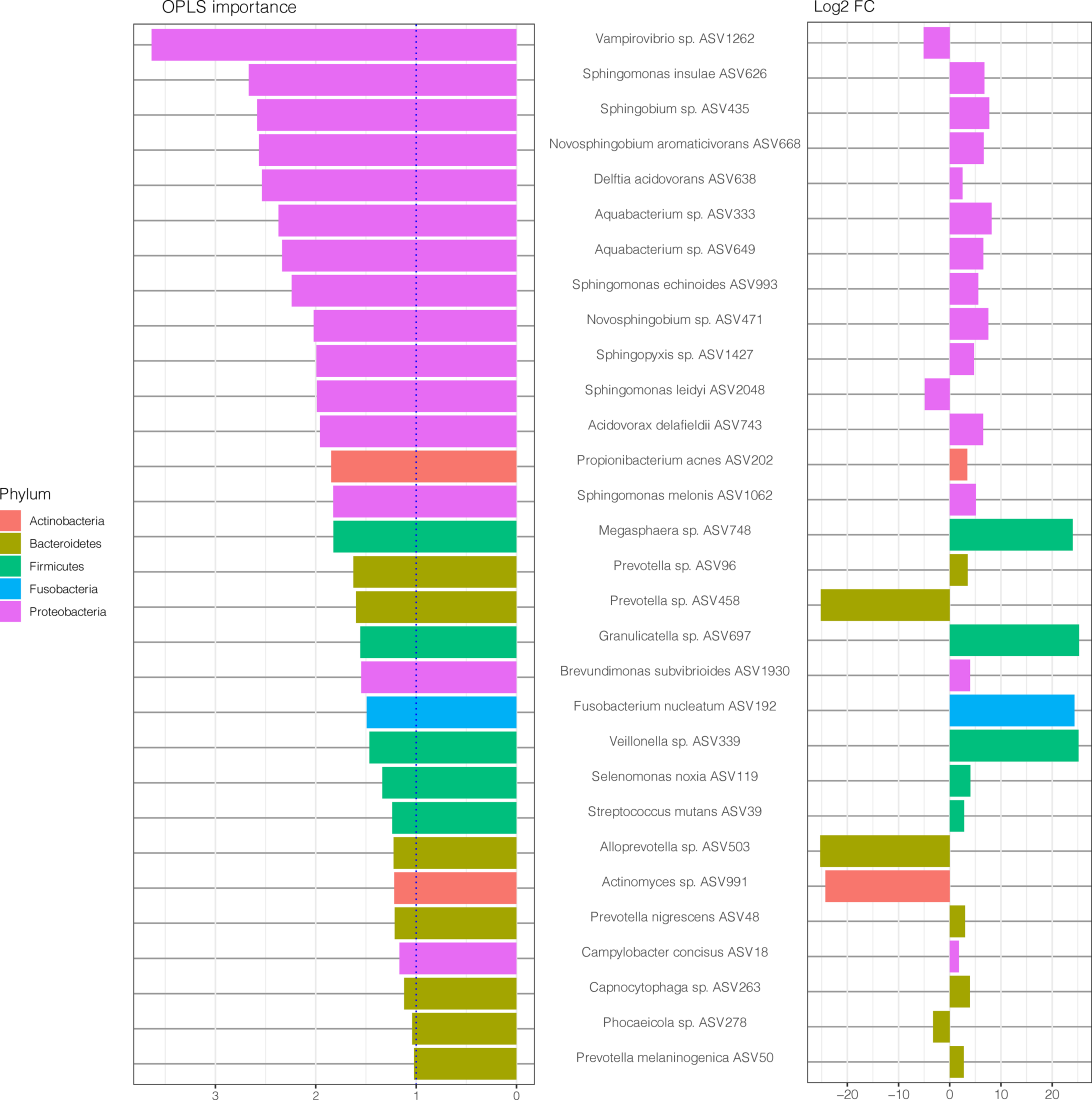
Amplicon sequences variants (ASVs) identified by OPLS-DA analysis with variable importance higher than 1 together with the log2 fold change (log2 FC), for ASVs with p value < 0.05, performed with size factor normalization of fold changes as implemented in DESeq2. The lowest annotated taxonomic rank is shown for each ASV as well as the ASV IDs used for the analysis.

To further explore the underlying biology of the classification and to juxtapose the two methods used (DESeq2 and OPLS-DA), we compared the most important ASVs for the classification of samples (OPLS-DA model, VIP > 1) and their corresponding log2 fold change (log2 FC) (DESeq2, p < 0.05), as demonstrated in **Figure 5**. Notably, when comparing this OPLS-DA with DESeq2 results (**Figure 2**), 30 of 36 ASVs with significant differential abundance (FDR adjusted p < 0.05) were identified as important ASVs (VIP >1) in the OPLS-DA model. Among these 30 ASVs, 50% were Proteobacteria, 23% Bacteroidetes, and 17% Firmicutes. Moreover, eight of the top 15 ASVs with the highest importance (**Figure 4**) had also a significant differential abundance (Log2 FC, p < 0.05), as shown in **Figure 5**. These included *Vampirovibrio* sp. ASV1262 having a positive correlation with non-RA group, and seven ASVs (*Sphingomonas insulae* ASV626*, Sphingobium* sp. ASV435, *Novosphingobium aromaticivorans* ASV668, *Delftia acidovorans* ASV638, *Aquabacterium* sp. ASV333, *Aquabacterium* sp. ASV649, and *Sphingomonas echinoides* ASV993) with a positive correlation with RA group.

### Classification of Samples From Periodontitis Patients With and Without RA-Based on ASVs and Inflammatory Mediators

To test whether the observed bacterial abundances and levels of host inflammatory mediators determined in the same saliva samples could be used to differentiate individuals with RA from those without RA, we also used an OPLS-DA model based on the ASVs and the levels of different inflammatory mediators. Totally, 71 samples consisting of 35 samples from non-RA and 36 samples from RA subjects, were used to train the model and cross-validation was performed (using training dataset) to evaluate the predictive power of the model. This model, based on combination of ASVs and inflammatory mediators, showed a better fit and prediction performance (R2Y value 0.96, Q2Y value 0.4520, **Supplementary Figure 1B**) than the OPLS-DA model with only ASVs. To further evaluate the predictive power of the model and to validate this model, the trained model was validated using the remaining 24 samples (12 non-RA, 12 RA). The model was able to classify the 24 samples into 12 non-RA and 10 RA subjects with high accuracy (0.917) and an AUC value of 0.9792.

### Association of Inflammatory Mediators With Microbiota

**Figure 6:**
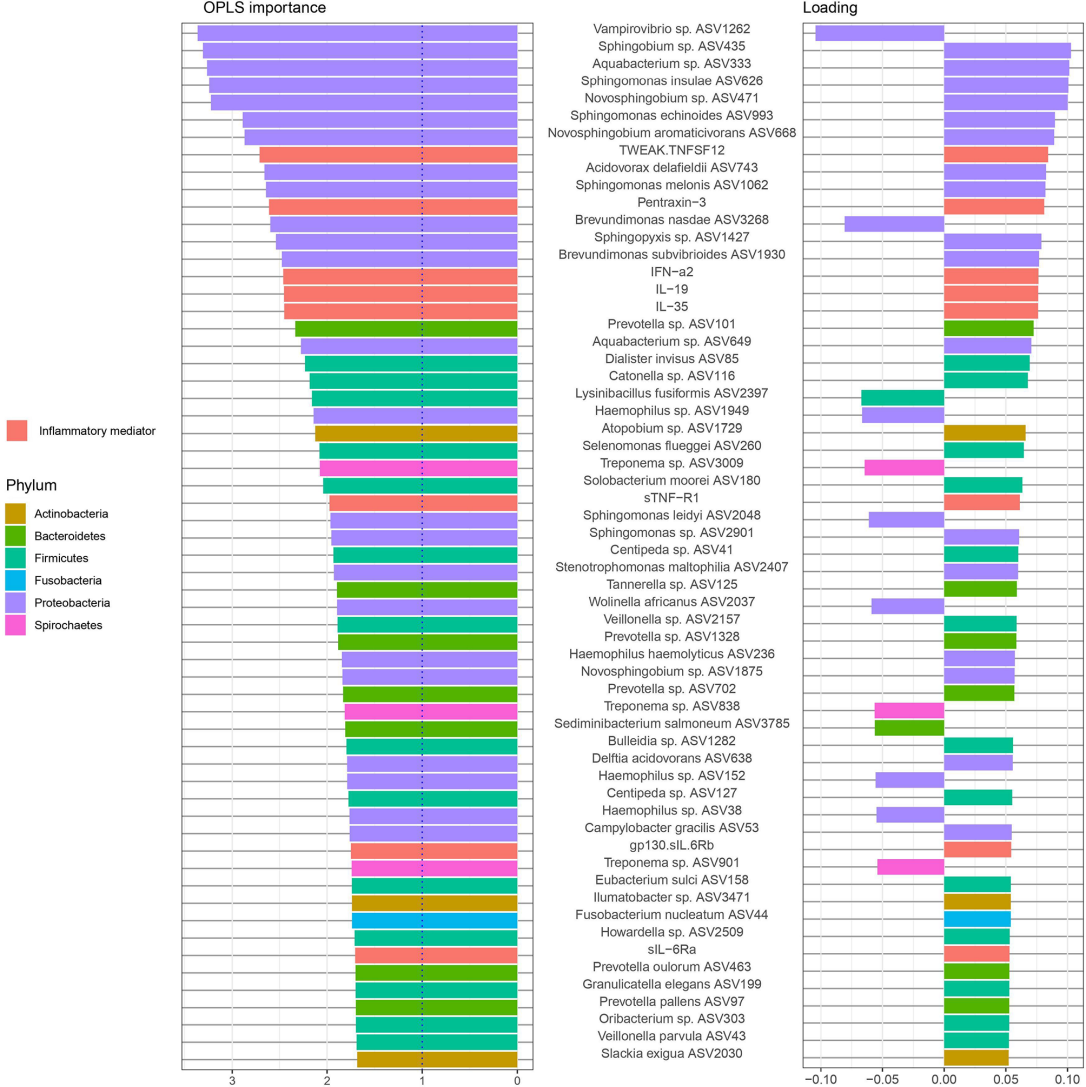
Amplicon sequences variants (ASVs) and inflammatory mediators/cytokines in saliva samples identified by Orthogonal Partial Least Squares Discriminant Analysis (OPLS-DA) analysis. In OPLS-DA, a single component serves as a predictor for the class. The sixty most important variables (with VIP > 1) for the classification of saliva samples from patients with and without RA are shown. The variable loading on the predictive component is shown (positive = discriminatory direction RA, negative= discriminatory direction non-RA). The lowest annotated taxonomic rank is shown for each ASV as well as the ASV IDs used for the analysis.

The ASVs and inflammatory mediators with the highest importance (OPLS-DA variable importance > 1) in the model are shown in **Figure 6**. The two variables with the highest importance were the ASVs *Vampirovibrio* sp. ASV1262 (positive correlation with periodontitis group with non-RA) and *Sphingobium* sp. ASV435 (positive correlation with periodontitis group with RA).

Among the twenty variables with the highest OPLS importance, five were cytokines (TWEAK/TNFSF12, Pentraxin-3, and IFN-α2, IL-19, and IL-35) positively correlated to RA samples and fifteen were ASVs. Majority of these ASVs, predominantly from the phylum Proteobacteria (11), Bacteroidetes (1) and Firmicutes (1), were found to be positively correlated to RA samples (positive loadings). Only two ASVs, *Vampirovibrio* sp. (ASV1262) and *Brevundimonas nasdae* (ASV3268) (both belonging to Proteobacteria phylum) were positively correlated to non-RA control samples (negative loadings).

**Figure 7:**
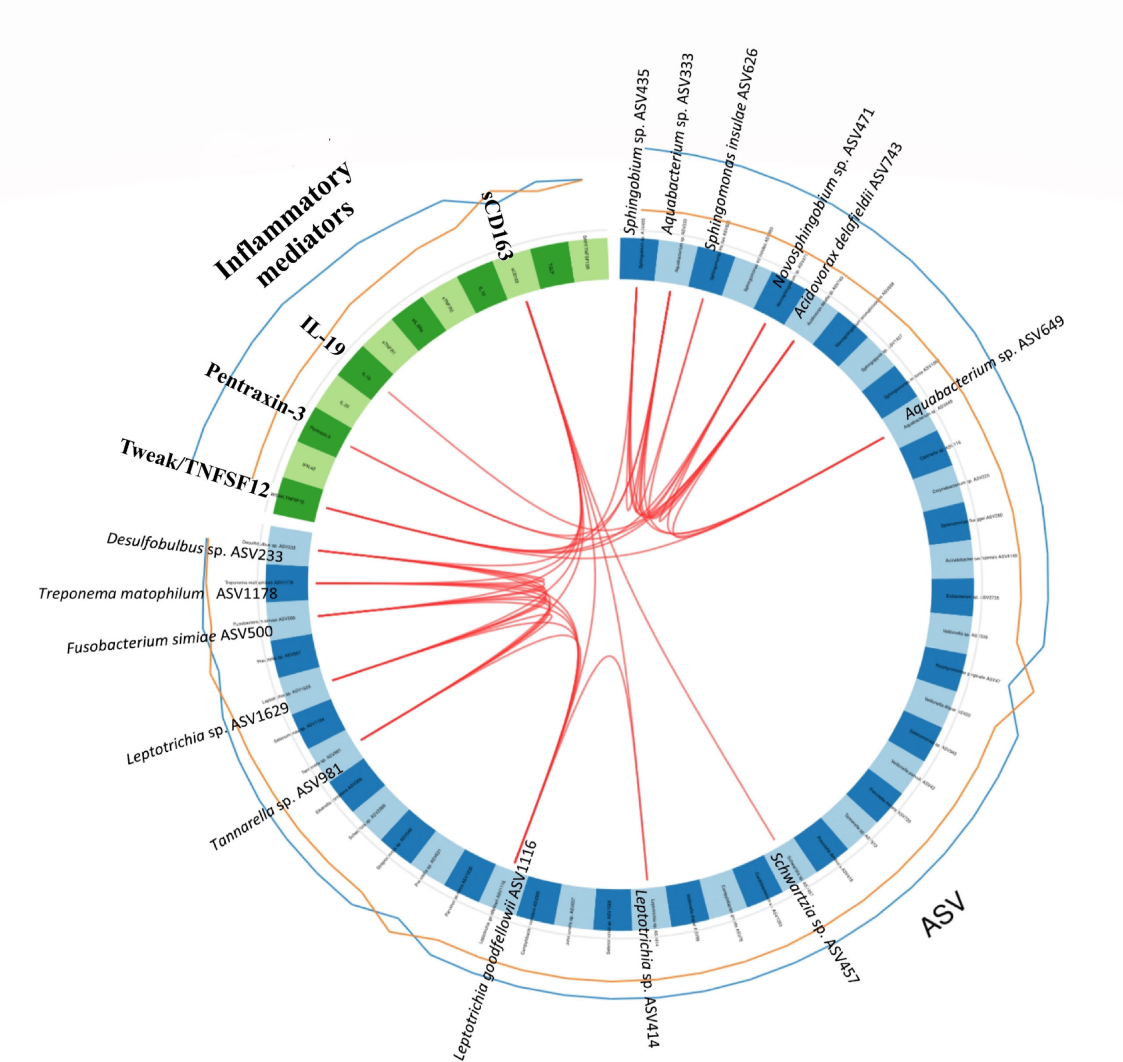
Circos plot depicting correlations between microbiota and inflammatory mediators/cytokines. The panel of Amplicon Sequence Variants (ASVs) and cytokines are identified by the sparse partial least squares discriminant analysis (sPLS-DA). The first and second component from the sPLS-DA model are included in the Circos plot. Blue variables represent ASVs and green variables represent inflammatory mediators/cytokines. The lines outside the circle represent the ASV abundance or the level of inflammatory mediators in samples from patients with RA (blue) and without RA (orange), respectively. Red lines inside the circle represent positive correlations between ASVs and host response mediators, at a correlation > 0.7. The lowest annotated taxonomic rank is shown for each ASV together with its ASV ID used.

To investigate correlations between the oral microbiota and corresponding host inflammatory mediators in RA, as well as possible combinations of microbiota and host response biomarkers, we performed the supervised method sparse partial least squares discriminant analysis (sPLS-DA). The interactions, identified between the ASVs and inflammatory mediators through the sPLS-DA is shown in a Circos plot (**Figure 7**). The plot revealed that, for the first and second component and at a cut-off value of 0.7, there was several correlations between the inflammatory mediators and ASVs. Among the significantly higher inflammatory mediators detected in the RA group (**Figure 3**), the TWEAK/TNFSF12 was identified as positively correlated with *Sphingobium* sp. (ASV435), *Aquabacterium* sp. (ASV333), *Novosphingobium* sp. (ASV471), *Acidovorax delafieldii* (ASV743), and *Aquabacterium* sp. (ASV649); the pro-inflammatory mediator pentraxin-3 with *Aquabacterium* sp. (ASV333), and *Acidovorax delafieldii* (ASV743); and IL-19 with *Acidovorax delafieldii* (ASV743) (**Figure 7**). All the above mentioned ASVs had significant Log2 FC >0 (DESeq2), high variable importance in projection (OPLS-DA model) and positively correlated to RA samples. Furthermore, sCD163, found to have significantly higher levels in the non-RA group, positively correlated with *Schwartzia* sp. (ASV457), *Leptotrichia* sp. (ASV414), *Leptptrichia goodfellowii* ASV1116, *Tannerella* sp. (ASV981), *Leptotrichia* sp. (ASV1629), *Fusobacterium simiae* (ASV500), *Treponema maltophilum* (ASV1178), and *Desulfobulbus* sp. (ASV233).

## Discussion

In the current study, we aimed to profile the oral microbiome composition and the host´s inflammatory mediators in saliva samples obtained from RA patients with periodontitis and non-RA subjects with periodontitis, as well as to investigate the different bacteria’s association with inflammatory mediators and their importance in classification of RA/non-RA-periodontitis, using the multivariate machine learning algorithms, OPLS-DA and sPLS-DA. Our results demonstrate that there indeed are two distinct oral microbial profiles for periodontitis patients with and without RA, based on the abundance of distinct ASVs, although not all the bacteria seem to be important for classification. The results further suggest that the combination of inflammatory mediators TWEAK/TNFSF12, pentraxin-3 and IL-19 and ASVs *Sphingobium* sp. (ASV435), *Aquabacterium* sp. (ASV333), *Acidovorax delafieldii* (ASV743), *Novosphingobium* sp. (ASV471), and *Aquabacterium* sp. (ASV649) could be used to distinguish periodontitis patients with RA versus without RA.

Periodontitis and RA are both multifactorial diseases characterized by chronic inflammation leading to tissue and bone destruction around teeth or joints, respectively. Although a relationship between these two diseases has been recognized for many years, the mechanisms and the pathophysiological processes linking the two diseases are still unknown (Lopez-Oliva et al., 2019; Bartold and Lopez-Oliva, 2020). The bacterial link between periodontitis and RA has been hypothesized and investigated for almost two decades due to the unique ability of oral pathogens, such as *P. gingivalis*, to be involved in the generation of citrullinated antigens and the subsequent production of anti-citrullinated protein antibodies (ACPAs) (Rosenstein et al., 2004; Wegner et al., 2010; Konig et al., 2016). ACPAs are specific predictive markers for the development of RA and have been associated with disease severity and joint destruction (Trouw et al., 2013). Recently, our group demonstrated that patients with RA had more moderate/severe periodontitis, and that this severe form of periodontal disease in RA was significantly associated with an altered subgingival microbial profile, and increased levels of systemic and oral inflammatory mediators (Eriksson et al., 2019). To further investigate this change in periodontal profile of RA-periodontitis patients, in this study we focused on describing the oral microbial and inflammatory mediator composition of periodontitis patients, with and without RA, in more detail and aimed to decipher the importance of different microbes and inflammatory mediators for classification and differentiation between the groups.

In our study, *P. gingivalis* was not included among the top 30 most important ASVs for the classification of samples and those with variable importance in projection (VIP) > 1. In agreement with our results, Kroese and colleagues reported that *P. gingivalis* was not identified as a discriminative zero-radius operational taxonomic units, zOTUs (Kroese et al., 2021). Furthermore, previous 16S rRNA gene sequencing studies, investigating the subgingival microbiome of patients with RA compared to osteoarthritis and healthy controls without periodontitis did not show subgingival compositions discriminating between RA and osteoarthritis (Mikuls et al., 2018), nor between RA and healthy controls regarding the periodontal pathogens *P. gingivalis* or *A. actinomycetemcomitans* (Lopez-Oliva et al., 2018). In addition, when comparing patients with established RA with no/mild periodontitis or moderate/severe periodontitis, our previous pilot study did not show any significant differences in the prevalence of *P. gingivalis* in subgingival plaque (Eriksson et al., 2019). Also, when using 16S rRNA gene sequencing, none of the pathogens currently implicated in citrullination (*P.gingivalis*, *Aggregatibacter actinomycetemcomitans* or *Cryptobacterium curtum*) were among the most abundant bacteria in saliva or subgingival plaque samples of RA patients. Thus, additional studies involving larger sample size of RA patients with periodontitis and controls with healthy periodontium will be needed to be able to detect differences in abundance of these citrullination associated pathogens.

To investigate the microbial differences between the groups in the current study, first a PCoA was performed revealing that salivary samples from patients with periodontitis clearly differed depending on RA status, with bacteria from phyla Actinobacteria, Bacteroidetes, Proteobacteria, and Firmicutes tending to be more common among the RA patients, whereas phyla Spirochaetes, Tenericutes and Chloroflexi were predominant in non-RA. The ASVs also differed in abundance depending on RA status in our study, with species from phyla such as Firmicutes, Fusobacteria and Proteobacteria being more abundant in RA, and species from phyla Bacteroidetes, Proteobacteria, and Actinobacteria dominating the abundance in saliva of non-RA patients with periodontitis. Using the detection of abundant microbes in samples of patients is a common approach to differentiate between different groups of patients in previous studies. Therefore, we wanted to investigate if the abundant ASVs in our study were also important for the differentiation between RA and non-RA periodontitis. To accomplish this, an OPLS-DA model as well as DESeq2 was used to investigate which ASVs were important for the classification of RA versus non-RA. The results showed that, when focusing on ASVs alone, ASVs from phyla Proteobacteria and Firmicutes were still among the most important microbes for classification of RA, although the topmost abundant species were no longer the most important in the model (**Figures 2**, **4** and **5**). Likewise, for non-RA samples, the most abundant ASVs were not the most important for classification, suggesting that it is the less abundant species that are most important for RA/non-RA classification. Notably, the species that were most important for the classification belonged to the same phyla, Proteobacteria and Bacteroidetes, as the top five most abundant microbes.

When studying the relationship between periodontitis and RA, inflammatory mediators are also considered important. Host response inflammatory mediators play an important role in the pathogenesis of chronic inflammatory diseases including periodontitis and RA, maintaining a chronic inflammation in addition to promoting autoimmunity that collectively contribute to tissue and bone destruction (Eberhardt and Fex, 1998; Hienz et al., 2015). Increased levels of several pro-inflammatory mediators have been well documented both in RA and periodontitis, and numerous cytokine-targeting therapies are successfully used in RA treatment (Nissila et al., 1983; Suarez-Almazor et al., 1994). Therefore, in this study we also determined the levels of inflammatory mediators to further investigate the microbial and host inflammatory interactions for classification of individuals with periodontitis, with and without RA. Our data showed that several inflammatory mediators were increased in RA-periodontitis saliva as compared to non-RA, including TWEAK/TNFSF12, pentraxin-3, IL-19, IL-35, gp130/sIL6Rb, sIL-6Ra, IFN-α2 and sTNF-R1. All these mediators, except sIL-6Ra, were also included as important mediators for RA classification based on OPLS-DA. Previous studies investigating the inflammatory mediators in serum of RA-patients, demonstrate a higher level of TWEAK, and Pentraxin-3 compared to controls (Park et al., 2008; Boutet et al., 2021). According to Boutet et al., the serum levels of Pentraxin-3 are correlated with disease activity and may act as a reliable marker of RA activity (Boutet et al., 2021). In addition, expression of IL-19 is also significantly elevated in synovial fluid of patients with RA compared to healthy controls, indicating an involvement of the regulation of synovial inflammation in patients with RA (Alanara et al., 2010).

Among the inflammatory mediators analysed in the current study, only sCD163, recognized to have anti-inflammatory potential (Frings et al., 2002), was significantly lower in saliva samples from patients with RA. This finding is in contrast to the serum levels of sCD163, reported to be elevated in patients with RA (with unknown periodontal status) compared to controls (Jude et al., 2013). One explanation for the differences between those serum and the saliva levels of sCD163, studied in our periodontitis affected study population, may be due to the immune response and inflammatory mediators locally released from the periodontium, which may affect the levels of CD163 in RA differently. This assumption is in line with the findings by Ayravainen et al., 2018 reporting that the concentrations of inflammatory biomarkers differ in serum and saliva from patients with RA. Moreover, the CD163 expression has been shown to be suppressed by proinflammatory mediators (such as TNF α and interferon family) (Buechler et al., 2000). Thus, the increased levels of several proinflammatory mediators detected in our study could have had an effect on the sCD163 levels detected in saliva. Notably, sCD163 did not associate with any ASVs important for RA or non-RA classification, which is in line with the findings by Jude et al., 2013 that no significant correlations could be found between serum sCD163 levels and disease activity of RA.

To test whether we could increase the accuracy and predictive performance of our RA/non-RA classification models, a combination of ASVs and inflammatory mediators was also investigated, this approach showed a better fit for classification (R2Y value 0.96, Q2Y value 0.4520, accuracy 0.917 and AUC 0.9792). Interestingly, two (*Vampirovibrio* sp. ASV1262 and *Sphingomonas leidyi* ASV2048) of the most enriched ASVs found in the saliva of non-RA periodontitis patients (**Figure 2**), were also highly important when combined with inflammatory mediators, indicating their relevance for the differentiation between the groups (**Figure 6**). In saliva samples from participants with RA, 11 of the most abundant ASVs were also highly important in the OPLS-DA model combining inflammatory mediators with ASVs; *Aquabacterium* sp. (ASV333), *Sphingobium* sp. (ASV435), *Novosphingobium* sp. (ASV471), *Sphingomonas insulae* (ASV626), *Aquabacterium* sp. (ASV649), *Acidovorax delafieldii* ASV743, *Sphingomonas echinoides* ASV993, *Sphingomonas melonis* ASV1062, *Sphingopyxis* sp. (ASV1427), *Brevundimonas subvibrioides* (ASV1930), *Delftia acidovorans* (ASV638) (**Figure 6**). All of these important ASVs, both in non-RA and RA group, belonged to the Proteobacteria phylum. Controversially, for non-RA, four of the most highly abundant and previously highly important ASVs (*Alloprevotella sp* ASV 503, *Prevotella* ASV 458, *Actinomyces* sp. ASV 991 and *Phocaeicola* sp. ASV278) were no longer among the most important for classification of non-RA when combined with inflammatory mediators in the new model (**Figure 6**). These results indicate, that although abundance of a specific bacteria might be used for disease classification, for e.g. in non-RA the highly abundant *Prevotella* sp. ASV 458 (**Figures 2** and **5**), a specific combination of microbes and inflammatory mediators can be even more useful in disease identification. Thus, the combination of ASVs and host response inflammatory mediators may be a better alternative to early predict the patients with RA.

To further investigate the interactions and correlations between the oral microbiota and host response biomarkers/inflammatory mediators, sPLS-DA and a Circos plot was performed. The results showed that five of the most highly enriched ASVs in RA and most important for RA classification (*Sphingobium* sp. ASV435, *Aquabacterium* sp. ASV333, *Novosphingobium* sp. ASV471, *Acidovorax delafieldii* ASV743, and *Aquabacterium* sp. ASV649) were all positively correlated with the most upregulated inflammatory mediators in RA saliva. All five ASVs correlated with TWEAK/TNFSF12, two of these (*Aquabacterium* sp. ASV333 and *Acidovorax delafieldii* ASV743) also correlated with pentraxin-3, and (*Acidovorax delafieldii* ASV743) correlated with IL-19. The ASVs mentioned above have not previously been associated with RA or periodontitis. However, it was recently reported that the salivary microbiome can differ structurally in its composition with regards to bacteria from the Proteobacteria phyla, including abundance of *Aquabacterium* sp., in individuals exposed to early-life adversity/stress as compared to controls (Charalambous et al., 2021). Moreover, in patients with early RA stressful life events have recently been reported to precede the onset of symptoms (Germain et al., 2021). Therefore, it is plausible that our findings of different salivary microbiome compositions (with regards to several ASVs from the Proteobacteria phylum) in combination with inflammatory mediators, may provide new insights into disease classification in patients with periodontitis, with and without RA.

Regarding the inflammatory mediators that were most highly upregulated in RA patients (IL-19, TWEAK/TNFSF12 and pentraxin-3) and positively correlated with the above mentioned ASVs, the cytokine IL-19 has previously been found to be highly expressed in the joints of patients with RA and therefore suggested as a potential target for therapy in RA patients (Alanara et al., 2010; Alghasham and Rasheed, 2014). Similarly, the levels of TWEAK/TNFSF12 have been reported as elevated in both synovial tissue and serum of patients with RA and found to correlate well with the disease activity of RA (Park et al., 2008; Dharmapatni et al., 2011). The last inflammatory mediator, pentraxin, that together with ASVs could distinguish between RA and non-RA patients with periodontitis, plays a central role in both infection and systemic inflammation (Qiu et al., 2021). The expression of this mediator is increased in serum/plasma and synovial fluid of RA individuals (Wu et al., 2020). Interestingly, in line with our findings, pentraxin-3 has recently been suggested as a novel biomarker for RA diagnosis and a sensitive indicator of clinical arthritic activity in RA when compared to conventional diagnostic marker C-reactive protein (Qiu et al., 2021). Pentraxin-3, has also been suggested to be a key biomarker for periodontal disease diagnosis (Kathariya et al., 2013), which is supported by our previous findings that salivary levels of pentraxin-3 in combination with specific ASVs (belonging to *Streptococcus* sp., *Selenomonas* sp., and *Treponema* spp.) could discriminate between patients with periodontitis and healthy controls (Lundmark et al., 2019). Nevertheless, the correlations between microbiota and expression of inflammatory mediators may be due to inflammation affecting the bacterial composition and could potentially contribute to the development and maintenance of the chronic inflammatory diseases.

The strength of our study includes the relatively large number of saliva samples from periodontitis patients with RA and controls without RA. Additional strength of the current study lies in its combination of investigating the microbial composition and its host inflammatory response applying the machine learning approach to predict biomarkers, of bacterial pathogens and/or inflammatory mediators, for classification of samples associated with periodontitis and RA. There are, however, some limitations with our study to be taken into consideration. First, we were not able to match the groups for gender since RA is two to three times more common in females than in males (Oliver and Silman, 2009), resulting in a higher ratio of women in the periodontitis group with RA as compared to periodontitis without RA. Given that males have been shown to be more prone to periodontitis (possibly due to poorer oral hygiene) (Natto et al., 2018) and that we lack information about oral hygiene measures, we consider this as a limitation of our study. On the other hand, the participants were matched for age, a well-known predictor and confounder in periodontitis research (Natto et al., 2018). This is an additional strength of our study investigating the interactions between host-inflammatory response and microbiota since age has been associated with molecular changes correlated with the immune response (Hajishengallis, 2010). Another limitation of the study was that we were not able to re-classify the diagnosis and severity of periodontitis according to the new classification (Caton et al., 2018) as the recruitment of participants was initiated before 2017, and we therefore lack detailed data to ensure a correct classification. Moreover, some of the study participants, from both RA and non-RA group, also had systemic diseases such as cardiovascular diseases and diabetes, indicated to be associated with periodontitis and dysbiosis of the microbiota in the oral cavity (Zhang et al., 2018; Cugini et al., 2021). For example, hyperglycemia may modify the composition of oral microbiota and, poor glycemic control has been associated with increased levels and occurrences of periodontal pathogens in the subgingival biofilm of subjects with type 2 diabetes (Miranda et al., 2017). However, it is not yet clear whether these changes are the consequence of hyperglycemia or the presence of periodontal diseases (Matsha et al., 2020). In addition, drugs used by the patients in order to control the different diseases may also influence the microbial composition and the diagnosis of periodontal disease, although research on medication use on the oral microbiome is limited. Thus, DeClercq and colleagues recently reported negligible effects of commonly used medications on microbial diversity and small differences in the relative abundance of specific taxa, suggesting minimal effect of commonly used medication on the salivary microbiome (DeClercq et al., 2021).

To our knowledge this is the first study investigating the classification and prediction of the oral microbiome and host’s inflammatory mediators to distinguish periodontitis patients with RA from those without RA. Previous studies have investigated the subgingival microbiome of patients with RA compared to subjects with osteoarthritis and healthy controls without periodontitis (Lopez-Oliva et al., 2018; Mikuls et al., 2018). In the current study, where both groups have periodontitis, will hopefully increase the possibility to identify the specific bacteria/microbes associated with RA. Based on investigations of abundance, importance for classification and correlations between ASVs and inflammatory mediators, our results suggest that inflammatory mediators TWEAK/TNFSF12, pentraxin-3 and IL-19 and ASVs *Acidovorax delafieldii* (ASV743), *Sphingobium* sp. (ASV435), *Aquabacterium* sp. (ASV333), *Novosphingobium* sp. (ASV471) and *Aquabacterium* sp. (ASV649) could be used to distinguish periodontitis patients with RA from those without RA. Furthermore, our results suggest that a combination of inflammatory mediators and microbes could potentially be more efficient for classification of RA and non-RA periodontitis, as compared to abundance of microbes alone, when using salivary samples.

## Data Availability Statement

The datasets presented in this study can be found in online repositories. The names of the repository/repositories and accession number(s) can be found below: https://www.ebi.ac.uk/ena, PRJEB21767.

## Ethics Statement

This study was approved by the Regional Ethical Review Board in Stockholm. The patients/participants provided their written informed consent to participate in this study.

## Authors Contributions

TY-L, AA, AL, KE and LD contributed to the study concept and design. KE, GF, LJ, and AC contributed to patient collection, sample collection and obtained clinical metadata. AL, YH, KE, and LL performed the experiments. AL together with LD performed the bioinformatics analysis. AL, TY-L, KE, and LD analyzed the data. KE, AL, LD, TY-L and CF wrote the manuscript. All authors, except AC (passed away 2021) revised and approved the final version of the manuscript.

## Funding

This study was supported by grants from Region Stockholm; the steering group KI/Region Stockholm for dental research (SOF); the Swedish Research Council (2017-02084); the Patent Revenue Fund for Research in Preventive Odontology; and Karolinska Institutet (Ulla and Gustaf af Uggla Foundation).

## Conflict of Interest

The authors declare that the research was conducted in the absence of any commercial or financial relationships that could be construed as a potential conflict of interest.

## Publisher’s Note

All claims expressed in this article are solely those of the authors and do not necessarily represent those of their affiliated organizations, or those of the publisher, the editors and the reviewers. Any product that may be evaluated in this article, or claim that may be made by its manufacturer, is not guaranteed or endorsed by the publisher.
