# Supplementary figures and images for: Salivary Microbiota and Host-Inflammatory Responses in Periodontitis Affected Individuals With and Without Rheumatoid Arthritis
Source: Front Cell Infect Microbiol. 2022 Mar 14;12:841139. doi: 10.3389/fcimb.2022.841139 (PMC8964114; doi:10.3389/fcimb.2022.841139)

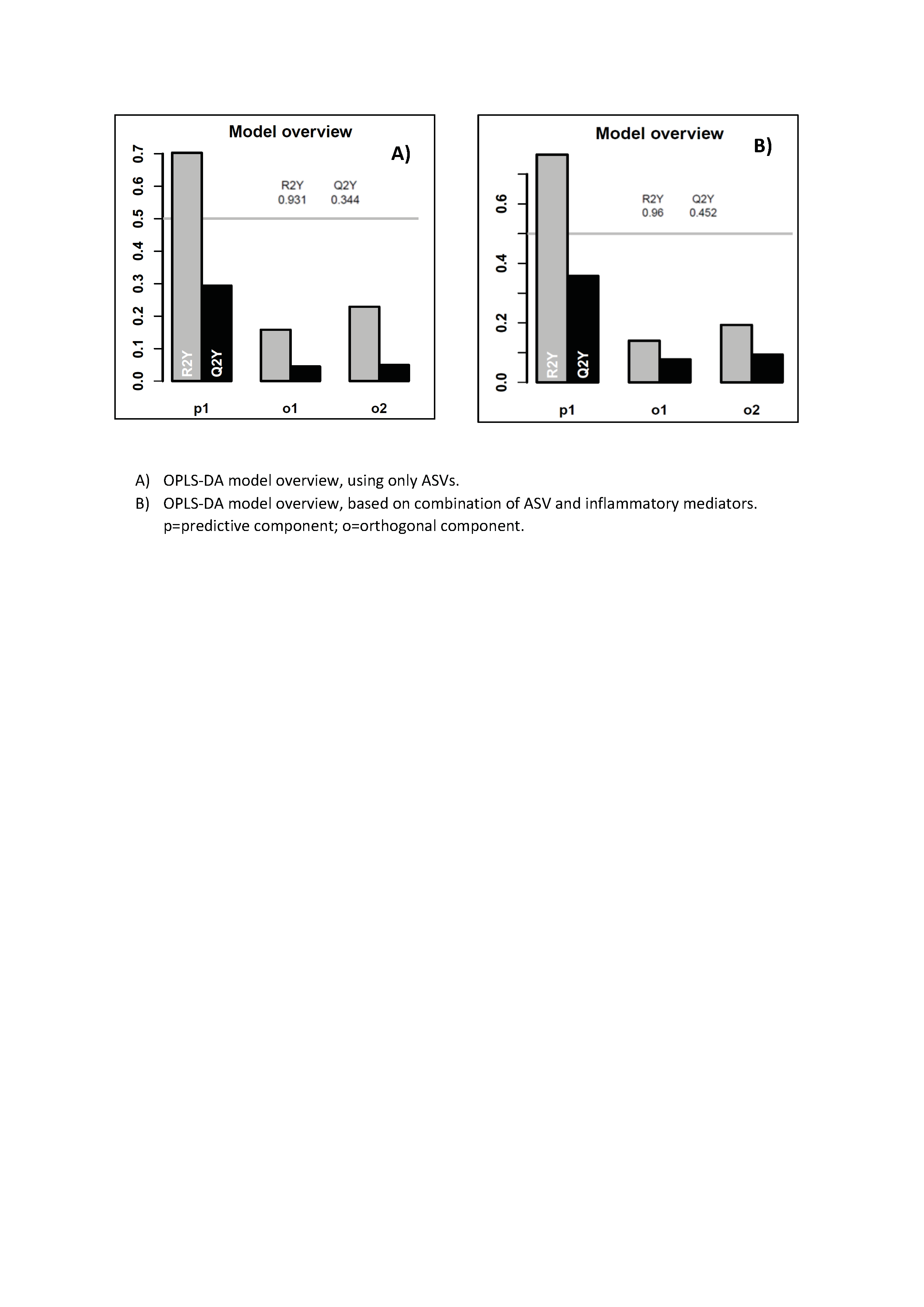

Supplement: Supplementary file 1 [file Image_1.tiff]
